# “Real world outcomes of intravitreal brolucizumab for persistent diabetic macular edema”

**DOI:** 10.1186/s40942-025-00708-y

**Published:** 2025-09-30

**Authors:** Saarang Hansraj, Ritesh Narula, Vishal Ramesh Raval, Raja Narayanan, Mudit Tyagi

**Affiliations:** https://ror.org/01w8z9742 grid.417748.9 0000 0004 1767 1636 Smt. Kanuri Santhamma Centre for Vitreo Retinal Diseases, Kallam Anji Reddy Campus, Anant Bajaj Retina Institute, L V Prasad Eye Institute, Hyderabad, India

**Keywords:** Persistent diabetic macular edema, Brolucizumab, anti-VEGF, Optical coherence tomography, Recalcitrant diabetic macular edema

## Abstract

**Aim:**

Chronic Persistent Diabetic Macular Edema can be a clinical challenge. The aim of this study is to assess the outcomes of intravitreal brolucizumab 6 mg/0.05 ml for persistent diabetic macular edema (P-DME) in a real-world clinical setting.

**Methods:**

A prospective interventional trial of consecutive patients with P-DME was conducted at a tertiary care center in India. P-DME was defined as edema persisting despite more than three intravitreal anti-VEGF injections or despite a combination of anti-VEGFs, intravitreal steroids and/or laser photocoagulation. The change in visual acuity (VA), central retinal thickness (CRT) and any incidences of adverse effects were analyzed.

**Results:**

19 eyes of 13 patients received a mean of 3.3 injections at a mean interval of 11.1 weeks. The median VA improved from 0.40 logMAR (20/50) to 0.35 logMAR (20/44) after a mean period of 13 weeks after the last injection, which was statistically significant (*p* = 0.004). The CRT reduced from 517 microns to 237 microns (*p* = 0.001). One patient had an episode of intraocular inflammation, which was treated successfully with topical steroids. The same patient again received brolucizumab with no recurrent inflammation.

**Conclusions:**

Intravitreal brolucizumab helped improve visual acuity in patients with P-DME and achieved a reduction of 54.1% in the CRT at a longer mean reinjection interval. The rate of intraocular inflammation was 5.2% without any permanent visual impairment.

## Introduction

Diabetic retinopathy (DR) is one of the leading causes of preventable blindness amongst individuals of working age group [1]. Diabetic macular edema (DME) represents a spectrum of retinopathy with accumulation of fluid and thickening of the macula. The treatment options for DME includes laser photocoagulation, intravitreal anti-vascular endothelial growth factors (anti-VEGFs), intravitreal steroids and vitrectomy [2]. While Anti-VEGFs have become the first line agents to treat center involving DME (CI-DME), some patients can have a persistence of retinal fluid termed as persistent DME (P-DME). There is no universally agreed upon term, and such patients may also be called as having recalcitrant, resistant or refractory DME [3]. 

Congruently there is no universally defined quantitative cutoff to identify P-DME. Studies have specified a variety of treatment schedules with sub-optimal responses before deeming a patient to have P-DME, such as poor response from 3 monthly consecutive anti-VEGF treatments [4] to partial response from 4 out of 6 injections [5]. Protocol T of the DRCR.net defined eyes having edema beyond the initial 24 week period to have chronic persistent DME.^6^ Protocol AC set the point of switching from Bevacizumab to Aflibercept at 12 weeks if edema was deemed ‘persistent’ [7]. 

Brolucizumab (Pagenax, Novartis Pharma AG, Basel, Switzerland) is a humanized single-chain antibody fragment with a molecular weight of ~ 26 kD [8]. 1 year results of the Kestrel and Kite studies showed that Brolucizumab 6 mg was non inferior to Aflibercept in mean change in BCVA from baseline, and more subjects achieved central subfield thickness (CST) < 280 μm, and fewer subjects had persisting subretinal and/or intraretinal fluid, with more than half of brolucizumab 6 mg subjects maintained on 12 weekly dosing [9]. Some inflammatory events were documented in the same trials and have also been reported from other studies [9–11]. Brolucizumab can be a valuable option in managing P-DME and we aimed to see its beneficial and adverse effects in our patient population in a real world setting.

## Methods

A retrospective analysis of consecutive patients with persistent CI-DME treated with intravitreal brolucizumab (6 mg/0.05 ml) was conducted between January 2022 and August 2023 at a tertiary care eye institute in South India, to assess the impact of the drug on visual acuity (VA) and central retinal thickness (CRT) in a real world clinical setting.

The study followed the tenets of the Declaration of Helsinki and was approved by the institutional review board (IRB-XXX). Written informed consent was taken from each patient.

P-DME was defined as:-.

1. CI-DME persisting a month after 3 monthly doses of the same intravitreal anti-VEGF injection.
2. CI-DME persisting a month after a combination of intravitreal anti-VEGF injections with focal or grid laser photocoagulation.
3. CI-DME persisting after a month after a combination of 3 monthly doses of intravitreal anti-VEGF injections with a switch to intravitreal steroid injection or intravitreal dexamethasone implant.
4. CI-DME persisting a month after treatment despite a switch between different anti-VEGF agents.
5. Any combination of the above.

All patients with P-DME who were treated with only intravitreal brolucizumab between January 2022 and August 2023 were included.

Patients who had macular pathologies besides CI-DME which could be responsible for decreased visual acuity were excluded. We did not include any patient with prior episodes of non-infectious intraocular inflammation in either eye. We also excluded all patients who failed to maintain regular follow up in the clinic.

The study was conducted in the out-patient department and patients with P-DME were treated on a pro re nata (PRN) basis. A subjective measurement of VA with the Snellen visual acuity chart and a comprehensive ocular examination was done at each visit. Optical coherence tomography (OCT) scan was performed at each visit, using either the Zeiss Cirrus HD OCT (Carl Zeiss Meditec, Dublin, CA), Heidelberg Spectralis OCT (Heidelberg Engineering, Heidelberg, Germany) and Topcon DRI Triton SS-OCT (Topcon, Tokyo, Japan) machines. Two OCT line scans perpendicular to each other were passed through the fovea and the mean foveal thickness was calculated to determine the CRT. Fundus fluorescein angiography (FFA) was performed depending on the clinical indication. The last visit included in the study was either the last visit within the study period or the first visit after the conclusion of the study period, if the final intravitreal injection was in the final month of the designed study. The decision to treat with the intravitreal drug at the visit or to observe was taken by retina fellowship trained ophthalmologists with more than a decade of experience.

All previous data prior to the study period was analysed which included visual acuity at first instance of DME, diabetic retinopathy status, OCT and fluorescien angiography, treatment modalities for DME, past surgeries, non diabetic ocular co-morbidities, systemic co-morbidities and any previous adverse reactions to any drug.

Patients were reviewed for signs of any adverse reaction to treatment. All patients in our hospital have an option of teleconsulting with the treating ophthalmologist and were advised to do so at any symptoms of pain, redness or sudden decrease in vision. Amongst the patients receiving brolucizumab in both eyes, the second eye was injected a week after the first eye was examined and checked for adverse reactions.

The patients were then asked to review back after a month. The decision to treat again with brolucizumab was assessed on the basis of visual gain and redution in severity of macular edema.

The clinical data of each patient was analysed with IBM SPSS Statistics^®^ software version 26 (IBM Corp, Armonk, NY, USA). A ‘p’ value p less than equal to 0.05 was considered statistically significant.

## Results

19 eyes from 13 patients were included in the study. The mean age of patients was 64.4 years (44 years to 73 years). Our cohort consisted of 7 males and 6 females.

Out of 19 eyes, 13 eyes also (68%) had proliferative diabetic retinopathy. 10 patients had hypertension (77%), 4 patients had chronic kidney disease (30.7%) while 4 patients had deranged serum lipid levels (30.7%).

These characteristics are summarized in Table 1.

**Table 1:** Demographic and systemic co-morbidities

Category	Features	Number of eyes (%)
**Mean age of presentation**	64.4 years (range, 44–73 years)	
**Sex**	Male	7 (53.8%)
	Female	6 (46.2%)
**Laterality**	Unilateral	7 (53.8%)
	Bilateral	6 (46.1%)
**Systemic co-morbidities**	Diabetes mellitus	13 (100%)
	Hypertension	10 (76.9%)
	Diabetic nephropathy	4 (30.7%)
	Dyslipidemia	4 (30.7%)
	Anemia	2 (15.3%)
	Coronary artery disease	2 (15.3%)
	Carotid artery disease	1 (7.6%)
	On insulin therapy	4 (30.7%)
	H/O percutaneous coronary artery angioplasty	1 (7.6%)

Amongst ocular co-morbidities, 2 eyes (10.5%) had disc pallor, 2 eyes (10.5%) had angle closure glaucoma and 1 eye (5.3%) had neovascular glaucoma, which in all three cases was adequately controlled.

18 of 19 eyes had received prior injections of intra-vitreal anti-VEGF agents (18/19, 94.7%). The intravitreal agents included 1.25 mg/0.5 ml Bevacizumab (Avastin; Genentech Inc., F. Hoffmann-La Roche AG, Basel, Switzerland), 0.5 mg/0.05 ml Ranibizumab (Lucentis; Novartis Pharma AG, Basel, Switzerland, and Genentech Inc., South San Francisco, CA, USA), 1.25 mg/0.05 ml Ziv-Aflibercept (Zaltrap; Regeneron, New York, USA) and 0.5 mg/0.05 ml biosimilar Ranibizumab (Razumab; Intas Pharmaceuticals Limited, Ahmedabad, India). The mean number of intravitreal injections per eye was 5.7 were received before the trial period. 6 eyes had also received a mean of 1.3 intravitreal steroid injections in the form of a dexamethasone drug delivery system (DDS) (Ozurdex; Allergan, Inc, Irvine, CA, USA) (6/19, 31.5%) as well as additional single intravitreal triamsinolone in 1 eye (1/19, 5.3%). 4 eyes (4/19, 21%) had also undergone laser treatment either as macular grid laser, focal laser therapy or micropulse laser.

13 eyes (68%) underwent pan-retinal photocoagulation. Out of 19, 12 eyes (63%) were pseudophakic, 6 eyes were cataractous (32%), of which none were visually significant and 1 eye (5%) had a clear lens.

The median best corrected visual acuity prior to starting brolucizumab was 0.40 logMAR (SD +/- 0.21 logMAR; 20/50 Snellen equivalent).

All patients received intravitreal brolucizumab over a period of 20 months. A total of 63 injections were given, with a mean of 3.3 injections per eye (1 to 6 injections per eye). The mean reinjection interval was 11.1 weeks (5.5 to 19 weeks). The mean period of follow up since the last injection was 13 weeks (4 to 36 weeks). The mean total period of follow up of the patients under our care from the diagnosis of DME to treatment with brolucizumab was 163.8 weeks (32 to 388 weeks).

The median best corrected visual acuity after treatment with brolucizumab was 0.35 logMAR (SD +/- 0.26; 20/44 Snellen equivalent).

Treatment details are summarized in Table 2.

**Table 2:** Description of cases with persistent diabetic macular edema (P-DME)

Eye	Age (years)	Sex (M/F)	Diabetic retinopathy treatment prior to brolucizumab	Anti-VEGF monotherapy (Y/*N*)	Number of anti-VEGF injections	Visual acuity at onset of trial	Central retinal thickness (CRT) at onset (microns) of trial	Number of injections of brolucizumab	Adverse effects (A/E)	Final visual acuity	Central retinal thickness (CRT) at last follow up (microns)
1	60	F	Anti-VEGFs, Ozurdex, Grid laser	N	4	20/30	265	6	Nil	20/30	175
2	60	F	Anti-VEGFs, Ozurdex, Grid laser	N	3	20/60	191	6	Nil	20/30	170
3	57	F	Anti-VEGF, IVTA, ozurdex, grid laser, focal laser, MPL, PRP	N	6	20/100	697	6	Nil	20/80	248
4	44	F	Ant-VEGF, ozurdex	N	2	20/40	253	2	Nil	20/25	299
5	44	F	Anti-VEGF	Y	3	20/100	347	5	Nil	20/25	271
6	64	M	Anti-VEGF, VR surgery	Y	3	20/100	674	1	Nil	LTFU	LTFU
7	64	M	Anti-VEGF	Y	3	20/60	655	1	Nil	LTFU	LTFU
8	62	M	Anti-VEGF, PRP	Y	4	20/50	227	2	Nil	20/60	150
9	62	M	Anti-VEGF, PRP	Y	6	20/50	208	4	Nil	20/50	162
10	51	F	Anti-VEGF, PRP	Y	6	20/40	457	5	Nil	20/50	500
11	51	F	Anti-VEGF, PRP	Y	4	20/40	517	5	Nil	20/30	376
12	52	F	Anti-VEGF	Y	4	20/40	507	2	Nil	20/20	221
13	55	M	Anti-VEGF, PRP	Y	4	20/125	889	1	Nil	20/125	689
14	73	F	Ozurdex, grid laser	N	0	20/60	666	5	IOI	20/30	141
15	70	M	Anti-VEGF, PRP	Y	7	20/50	685	2	Nil	20/40	243
16	64	M	Anti-VEGF	Y	5	20/50	550	2	Nil	20/30	513
17	47	F	Anti-VEGF, VR surgery, PRP	Y	4	20/60	272	2	Nil	20/40	213
18	68	M	Anti-VEGF, PRP, trabeculectomy	Y	17	20/320	713	3	Nil	20/160	589
19	68	M	Anti-VEGF, ozurdex, PRP	N	20	20/40	713	3	Nil	20/60	649

PRP: Pan-retinal photocoagulation, IVTA: Intravitreal triamsinolone, MPL: Micropulse laser, IOI: Intraocular inflammation

The median CRT at initiation of treatment with brolucizumab was 517 microns (SD +/- 217 microns, mean 499 microns). At end of the study, the median CRT was 237 microns (SD +/- 184 microns, mean 347 microns) with a reduction of 280 microns (54.1%). At final follow up, 5 eyes (26.3%) had complete resolution of the macular edema.

The reduction in macular edema was significant after the first injection. 17 eyes were seen within 2 months of the first dose and the median CRT was 243 microns. The median thickness prior to therapy in these eyes was 507 microns, thus amounting to a reduction of 52%.

Upon initiating therapy with brolucizumab, the OCT features included hyper-reflective foci (HRF) (*n* = 19, 100%), hard exudates (*n* = 13, 68.4%), disorganized retinal inner layers (DRIL) (*n* = 8, 42.1%), disrupted sub-foveal outer retinal bands (*n* = 7, 36.8%), epiretinal membrane (ERM) (*n* = 5, 26.3%) and SRF (*n* = 1, 5.2%).

At the last follow up the OCT features included HRF (*n* = 17, 89.4%), intra-retinal fluid (IRF) (*n* = 14, 77%), hard exudates (*n* = 9, 47.3%), DRIL (*n* = 8, 42.1%), disrupted sub-foveal outer retinal bands (*n* = 6, 31.5%) and an epiretinal membrane (ERM) (*n* = 4, 21%).

**Fig. 1:**
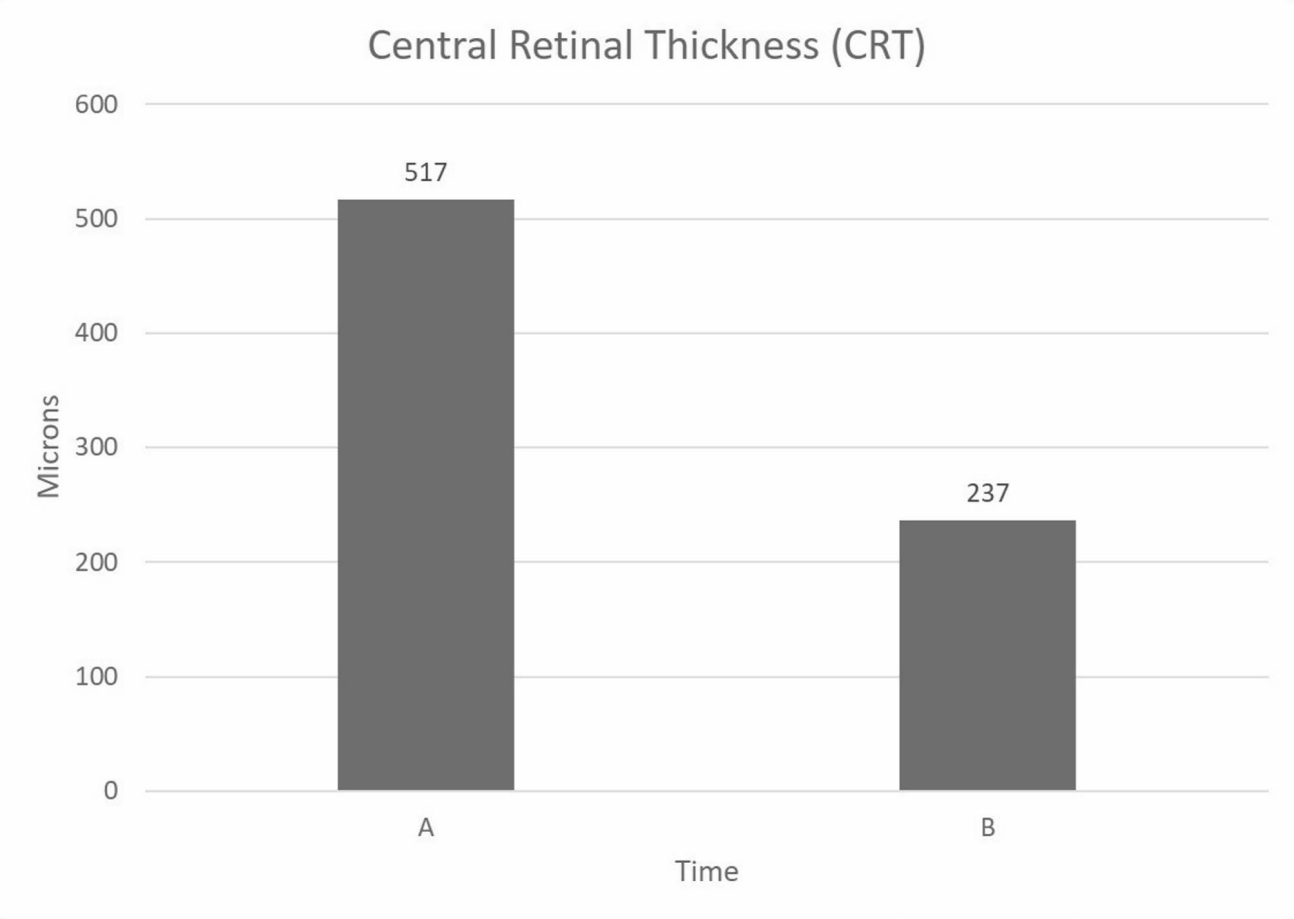
(**A**) Median macular thickness upon initiating therapy with brolucizumab and (**B**) Median macular thickness at last follow up. The median macular thickness shows a significant reduction of 54.1% at last follow up

The change in CRT has been demonstrated in Fig. 1.

During the trial one patient had an episode of acute non granulomatous iridocyclitis with intermediate uveitis. Not a single episode of endophthalmitis, vitreous hemorrhage, lens touch or retinal detachment occurred.

**Fig. 2:**
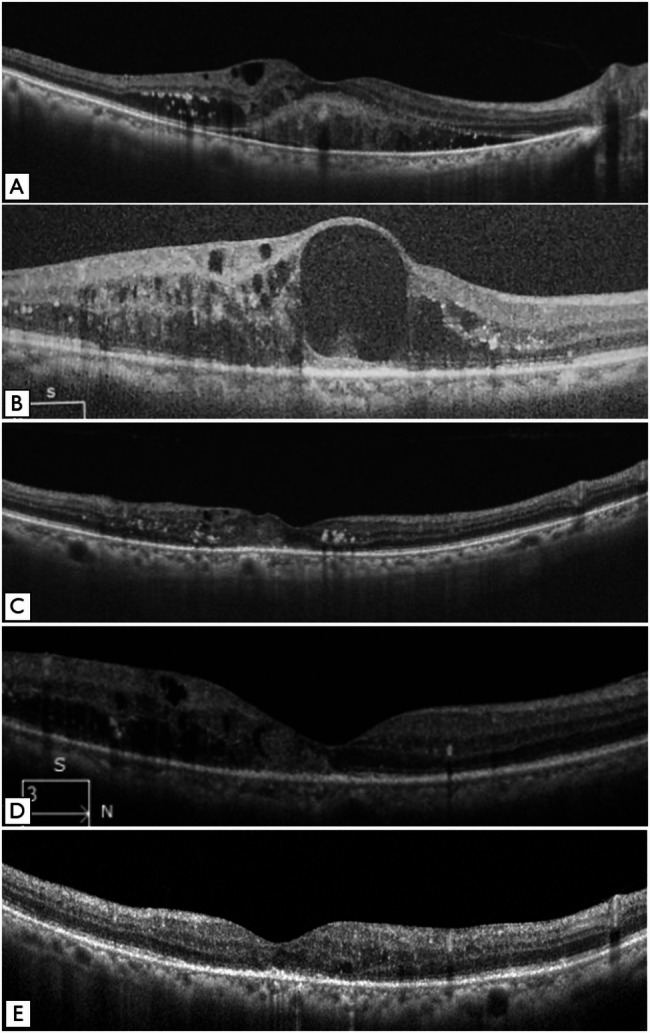
(**A**) The horizontal line scan of the spectral domain optical coherence tomography scan passing though the fovea shows center involving diabetic macular edema, along with intra and subretinal hard exudates (Topcon DRI Triton SS-OCT). The visual acuity at this visit was 20/60. The patient was treated with 4 injections of dexamethasone drug implant (Ozurdex) as well as macular grid laser. (**B**) After therapy patient had persistent macular edema with visual acuity of 20/60 and a decision was taken to treat her with intravitreal brolucizumab (Zeiss Cirrus HD OCT). (**C**) 2 months after the first injection she had improvement in visual acuity to 20/40 and a significant reduction in the macular edema as seen on the spectral domain optical coherence tomography line scan (Topcon DRI Triton SS-OCT). The intravitreal injection was repeated. It was at this point when she developed intraocular inflammation. She was treated with topical steroids, the inflammation resolved over 5 weeks and vision improved to 20/25. (**D**) 9 weeks after the 2nd injection patient presented with decrease in vison to 20/40. There was no recurrence of in flammation and the spectral domain optical coherence tomography line scan showed presence of intra-retinal fluid (Zeiss Cirrus HD OCT) and she was injected with brolucizumab for a third time. Over the course of the next 8 months she received additional two injections of brolucizumab. (**E**) At her last follow up she had a visual acuity of 20/20, with resolution of the macular edema, as seen on the enhance depth optical coherence tomography line scan (Heidelberg Spectralis OCT)

The onset of intraocular inflammation in our patient occurred 5 days after she received her 2nd intravitreal injection. She presented to the emergency clinic with complaints of an acute decrease in vision associated with pain and redness. Her visual acuity had dropped from 20/40 to 20/100. There was 2 + AC reaction, with diffusely distributed non granulomatous keratic precipitates and grade 1 vitritis. The retinal details could be appreciated till the third order vessels, with no vasculitis and/or vascular occlusion. The patient was treated with intense topical steroids. All the inflammation resolved, and the patient again received intravitreal brolucizumab. In total, she received 5 injections of intravitreal brolucizumab, and has been followed up for 9 weeks since her last injection. No further episode of inflammation recurred in the same eye. Her best corrected visual acuity at the time of last follow up was 20/20, with no macular edema. The serial changes in macular edema for this patient has been illustrated in Fig. 2.

The normality of the data was assessed by the Shapiro-Wilk test.

The median visual acuity prior to starting brolucizumab therapy was 0.40 logMAR (SD +/- 0.24 logMAR; 20/50 Snellen equivalent). The final median visual acuity was 0.35 logMAR (SD +/- 0.26 logMAR; 20/44 Snellen equivalent) for a gain of 0.05 logMAR. This difference in visual acuity was found to be statistically significant (*p* = 0.004) by the Wilcoxon Signed Rank test.

The median CRT of the 19 eyes included was 517 microns (SD +/- 217 microns, mean 499 microns), while final median CRT was 2237 microns (SD +/- 184 microns, mean 347 microns) for a reduction of 280 microns, which was significant (*p* = 0.001) by the Wilcoxon Signed Rank test.

The 52% reduction in CRT seen after the first injection was also found to be statistically significant (*p* = 0.001). However after the 1st injection the median visual acuity was found to be 0.39 logMAR, and this difference was found to be non-significant (*p* = 0.09).

The statistical analysis is provided in Table 3.

**Table 3:** Statistical outcomes

	Initiation of trial	Last follow up	*P* value
Mean visual acuity (logMAR)	0.48	0.37	
Median visual acuity (logMAR)	0.40	0.34	0.004
Interquartile range (logMAR)	0.40	0.30	
Standard deviation (logMAR)	0.24	0.26	
Mean Central subfield thickness (microns)	499	347	
Median Central subfield thickness (microns)	517	237	0.001
Interquartile range (microns)	420	300	
Standard deviation (microns)	217	184	

## Discussion

P-DME is a well-known, yet poorly defined entity [3]. It was shown in the Protocol T study by the DRCR.net group that after 3 consecutive monthly injections at week 12, DME persisted in 50.8, 72.9, and 53.2% of eyes that received aflibercept, bevacizumab, and ranibizumab, respectively [6]. 

The smaller size and higher concentration of brolucizumab are hypothesized to allow for a high degree of ocular tissue penetration [12]. 

With this information in mind we conducted a real world clinical study at our institute. Both protocol T [13] and I [14] have shown that that poor visual acuity gain at 12 week (post 3 monthly intravitreal injections) is a statistically significant indicator of lesser degree of gain in visual acuity at 2 years [6]. We have set this cut off period of monotherapy to select patients for our study. Early identification of patients with partial or poor response to anti-VEGF agents is of paramount importance to consider other treatment options in a timely manner.

Amongst our cohort only three eyes had received just three anti-VEGF injections prior to the study, with all cases showing a poor response to the initial treatment. All remaining patients had either received a greater number of anti-VEGFs or a combination of anti-VEGF, intravitreal steroid and/or laser photocoagulation therapy. (Table 2)

Over a period of 20 months we enrolled 19 eyes from 13 patients for our study which was conducted at a single referral center in south India. A significant improvement in both median visual acuity and median CRT was seen. The median visual acuity improved from 0.40 logMAR to 0.35 logMAR, which was even better than the median visual acuity of 0.40 logMAR when the patients first presented to us. The CRT also reduced significantly from 517 microns at initiation of the study to 237 microns for a reduction of 54.1%. It was seen in our study that the reduction in macular thickness or the “drying” effect of the drug preceded the improvement in visual acuity.

Despite the post hoc analysis of Protocol T showing that eyes with persistent edema were comparable to eyes without persistent edema in terms of VA at 2 years [6], there have been other studies which have showed that persistence leads to poorer visual outcomes. This is hypothesized to occur due to retinal microstructural changes and neuronal damage. Crucially unlike clinical trials, in a real world clinical scenario, it is unreasonable to expect patients to be compliant to an unspecified number of intravitreal injections for an indefinite amount of time. With passage of time and increased amount of interventions, patients are liable to drop out, be lost to follow up and suffer permanent visual loss [15]. 

Studies have shown a good response on switching therapy to either an intravitreal dexamethasone implant [16] or to another anti-VEGF agent [17]. Switching early to aflibercept has shown better results than switching late [18]. However the effects of switching to brolucizumab are limited. The impact of switching to brolucizumab early in the course of the disease seems to be similar to more traditional anti-VEGF agents [19]. Our study has shown that switching later in the course of the pathology when DME is persistent is also beneficial.

In addition to providing stabilization of vision, the drying effect seen in with brolucizumab was also significant in our study. Such a phenomenon has also been shown in treatment naïve eyes comparing brolucizumab to aflibercept [20]. A correlation between functional and anatomic parameters in DME remains incompletely defined. It is also a dilemma faced by clinicians whether to treat the changes in macular thickness or aim for gains in visual acuity [21]. Our study does not advocate for aggressive drying of the macula, but rather has shown that brolucizumab does do so significantly. During the entire PRN regimen followed by us, the decision when to treat versus when to inject took both the deterioration in visual acuity and increase in macular thickness into consideration. In a real world clinical scenario brolucizumab will provide faster and greater anatomical stabilization with lesser number of injections and lesser number of patient visits to the clinic. This anatomical stabilization may then provide protective cover for functional improvement in eyes with persistent DME.

A concern with brolucizumab has been its association with intraocular inflammation, retinal vasculitis and retinal vascular occlusion [11]. This has been seen in the pivotal KITE and KESTREL as well as the HAWK and HARRIER studies [22]. 

The median time to develop inflammation was found to be 25 days after the preceding injection and that for 98.0% (49/50) of patients, the first IOI-related adverse event was detected ≤ 56 days after the preceding injection. In almost all cases of vasculitis or vascular occlusion, signs of intraocular inflammation are seen [23, 24]. 

In our cohort we had one patient who developed intraocular inflammation without vasculitis. This occurred 5 days after receiving her 2nd dose of brolucizumab. She responded well to topical steroids, with no permanent sequelae. She was injected three times more with brolucizumab, with no episodes of inflammation. At last follow up she had compete resolution of macular edema, no intraocular inflammation and a vision of 20/20.

Thus over 20 months in 19 eyes of 13 patients, we encountered an adverse drug reaction in 1 eye (1/19 = 5.2%).

A strength of our study include the variety of patients included. All manner of DME patients either with non-proliferative or proliferative diabetic retinopathy, and patients with all available past treatment modalities for DME have been included.

The biggest strength of the study is undoubtedly its real world nature. Brolucizumab is a new drug, with most of its data and experience derived from meticulous well controlled trials. The real world impact is less well known and this lacuna is filled by our study. Our patients also had a few other non-retinal causes of decreased visual acuity, which may have impacted the results. However this is unavoidable in a real world scenario, and therefore these patients were not excluded from the study. We however were prudent in choosing whether such patients were offered brolucizumab therapy itself. Such decisions are taken by clinicians on a daily basis.

Our study is limited by its small sample size and small period of follow up. Patients also followed up irregularly at times limiting a more intense treatment regime, which could have yielded better outcomes.

## Conclusion

Our prospective interventional study has shown that intravitreal brolucizumab provides significant improvement in visual acuity and decrease in macular thickness in patients with P-DME, with the decrease in edema preceding the vision gain. While complete resolution of the edema was found only in 5 eyes (26.3%), the improvement in visual acuity was significant. Intraocular inflammation was seen in 1 eye (5.2%) which was treated with topical therapy with no permanent impact on visual acuity. We found the reinjection interval was 11.1 weeks, and in a real world setting where the challenges for patients to maintain compliance and afford treatment is exacting, brolucizumab provides a ray of hope for patients troubled with persistent diabetic macular edema.

## Data Availability

No datasets were generated or analysed during the current study.

## Declarations

Written informed consent was obtained from each participant of the study.
